# Coherence synthesis in nonlinear optics

**DOI:** 10.1038/s41377-025-01749-6

**Published:** 2025-02-26

**Authors:** Zihao Pang, Ady Arie

**Affiliations:** School of Electrical Engineering, Iby and Aladar Fleischman Faculty of Engineering, Tel Aviv University, Tel Aviv, 69978 Israel

**Keywords:** Nonlinear optics, Imaging and sensing

## Abstract

It is commonly assumed that nonlinear frequency conversion requires lasers with high coherence; however, this assumption has constrained our broader understanding of coherence and overlooked the potential role of incoherence in nonlinear interactions. In this work, we study the synthesis of optical spatial coherence in second harmonic generation using quadratic nonlinear photonic crystals. We demonstrate a method where the second harmonic coherence is customized by employing quantitative phase retrieval and a complex square-root filter sequentially on fundamental frequency speckles. As a proof-of-concept, we experimentally show incoherent imaging of a smiley face transitioning from infrared to visible light. Moreover, we apply this method to produce two representative types of structured light beams in second harmonic generation: incoherent vortex and Airy beams. During the nonlinear synthesis of incoherent vortex beams, we have, for the first time, experimentally verified the conservation of orbital angular momentum in the nonlinear frequency conversion process of a low-coherence source. Furthermore, the generated second-harmonic incoherent Airy beam preserves the self-acceleration characteristics of its fundamental frequency counterpart, remaining unaffected by reductions in coherence. Our results not only deepen the fundamental understanding of optical coherence but also unlock exciting possibilities for applications in infrared imaging and fluorescence microscopy where optical nonlinear interactions play an important role.

## Abstract (web summary)

Nonlinear synthesis of spatial coherence using second-order nonlinear photonic crystals. The coherence induced by the smiley face is synthesized in the far field of the crystal.

## Graphical abstract

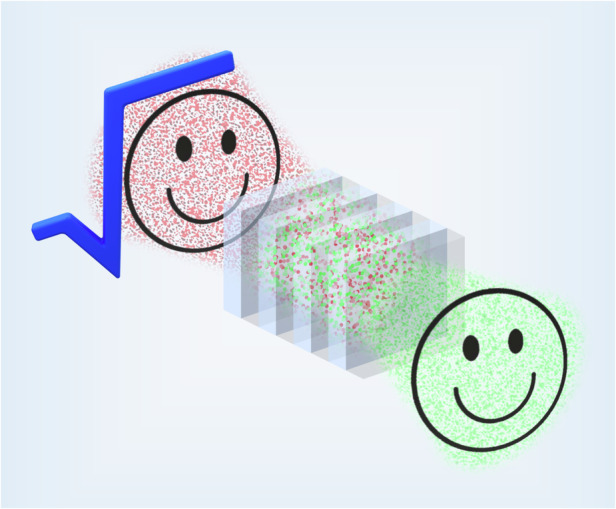

## Introduction

Coherence^1,2^ in optical systems is a fundamental concept that determines the phase stability and uniformity of light waves. It underpins a wide array of technologies and scientific investigations, ranging from the clarity of everyday photography to the resolution of advanced optical microscopy. The ability to understand and harness the coherence of light is crucial not just for practical applications such as optical coherence tomography^3–6^ widely used in medical diagnostics to create detailed images of tissues, but also for fundamental research in physics and engineering including quantum communication^7^ and computing^8,9^. In light-matter interactions, coherence dictates the behavior in a myriad of scenarios^10^ based on its presence or absence.

Nonlinear optics, since the invention of lasers^11^, has traditionally emphasized the critical role of highly coherent light sources for their effective manipulation in light-matter interactions. Although this focus has broadened the range of laser frequencies, primarily centered around achieving phase-matching conditions essential for efficient optical harmonic generation through nonlinear photonic crystals^12,13^, it potentially overlooks the vast landscape where incoherent or partially coherent light interacts with matters in equally profound ways. Recent advancements^14–18^ challenge this conventional mindset by demonstrating the potential of partially coherent or even incoherent light in nonlinear optics. For instance, using spatial light modulators, researchers have synthesized the desired spatial coherence in *χ*^(3)^ nonlinear crystals with self-focusing nonlinearity to tailor the elliptical beam shape with arbitrary orientations^14^. Similarly, *χ*^(2)^ nonlinear media present compelling avenues for advancing coherence control^19–22^, particularly in second harmonic generation, where the ability to shape the spatial coherence of light has profound implications. For instance, in microscopy^23^, precise manipulation of spatial coherence across a range of frequencies of light could possibly enable researchers to achieve higher resolution with respect to coherent systems, facilitating prolonged and more accurate observations of dynamic processes within living organisms. However, the dynamics of spatial coherence in nonlinear optical systems can be extremely non-trivial–especially in nonlinear frequency conversion systems–and the precise modulation on coherence structure is poorly understood. The first theoretical framework^19^ describing the evolution for the full four-dimensional spatial coherence function in *χ*^(2)^ nonlinear crystals, developed four decades ago, primarily focused on scenarios where the pump source exhibits Gaussian statistical properties. Until now, however, there has not been a systematic and comprehensive experimental scheme that can effectively customize the coherence of new-harmonic light in *χ*^(2)^ nonlinear materials due to the inherent complexity of the theoretical models.

**Fig. 1:**
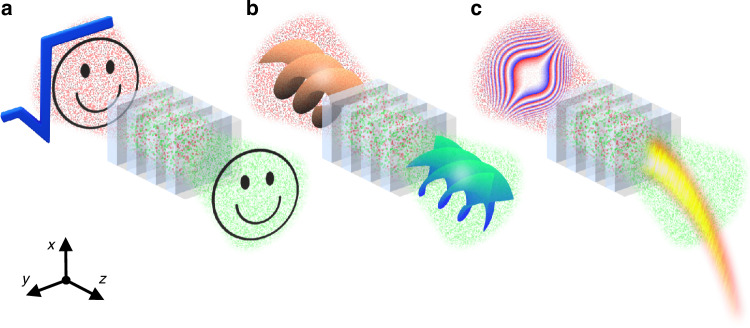
Nonlinear synthesis of spatial coherence with *χ*^(2)^ nonlinear photonic crystals. **a** Incoherent imaging of a smiley face transitioning from infrared to visible light. **b** The second harmonic incoherent vortex beams carrying 4*ℏ* orbital angular momentum (OAM) are nonlinearly generated from the fundamental frequency ones carrying 2*ℏ* OAM. **c** The second harmonic incoherent Airy beams that preserve the identical self-acceleration as the fundamental frequency incoherent Airy beams are nonlinearly generated

Here, we propose an alternative solution for the experimental synthesis of spatial coherence in second harmonic generation. The desired second-harmonic coherence is obtained by sending the carefully designed fundamental frequency speckles through a periodically poled nonlinear crystal, as shown in Fig. 1. This nonlinear synthesis of coherence is versatile for various purposes. As shown in Fig. 1a, we demonstrate this method experimentally by synthesizing the second harmonic coherence that replicates the smiley-face-induced coherence at the fundamental frequency, helping stimulate the new schemes for incoherent imaging of arbitrary shapes from the infrared to visible light spectrum. Moreover, we show the capability of nonlinear shaping of incoherent structured light beams by demonstrating the second harmonic generation of incoherent vortex (Fig. 1b) and Airy (Fig. 1c) beams. As we will see, the approach proposed here unlocks an additional degree of freedom in nonlinear beam shaping, going beyond the traditional modulation for deterministic physical quantities of light, such as amplitude and phase, to encompass the manipulation of stochastic ones like the spatial coherence. In principle, this same strategy for nonlinear synthesis of coherence can be deployed in various platforms such as nonlinear metamaterials^24^.

## Results

### Nonlinear synthesis of object-induced coherence

**Fig. 2:**
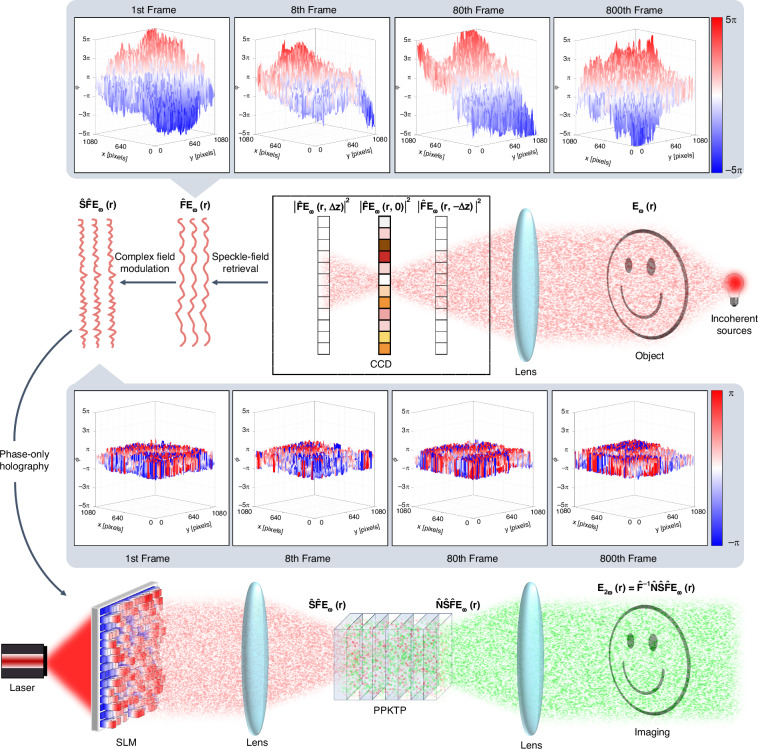
Schematic of object-induced coherence in nonlinear frequency conversion. A fully incoherent light source, consisting of 800 frames of random speckles, illuminates a smiley face at the fundamental frequency. The post-smiley speckles ${{\bf{E}}}_{{\boldsymbol{\omega }}}\left({\bf{r}}\right)$ are retrieved in the far field as complex fields $\hat{{\bf{F}}}{{\bf{E}}}_{{\boldsymbol{\omega }}}\left({\bf{r}}\right)$ with true continuous phase. This field undergoes amplitude and phase modulation to form fields $\hat{{\bf{S}}}\hat{{\bf{F}}}{{\bf{E}}}_{{\boldsymbol{\omega }}}\left({\bf{r}}\right)$. These frames of processed speckles are integrated into multiple holograms displayed on a spatial light modulator (SLM). A coherent fundamental frequency laser passing through the SLM reproduces the fields $\hat{{\bf{S}}}\hat{{\bf{F}}}{{\bf{E}}}_{{\boldsymbol{\omega }}}\left({\bf{r}}\right)$ on the incident plane of the nonlinear crystal **PPKTP**. At the crystal’s output plane, the resultant second-harmonic fields $\hat{{\bf{N}}}\hat{{\bf{S}}}\hat{{\bf{F}}}{{\bf{E}}}_{{\boldsymbol{\omega }}}\left({\bf{r}}\right)$ emerges. Finally, the coherence induced by the smiley face is synthesized in the far field of the crystal, comprising a series of second harmonic speckle fields denoted as ${\hat{{\bf{F}}}}^{-1}\hat{{\bf{N}}}\hat{{\bf{S}}}\hat{{\bf{F}}}{{\bf{E}}}_{{\boldsymbol{\omega }}}\left({\bf{r}}\right)$

Let us first consider the scene shown in Fig. 2 where a smiley face served as the object is illuminated by a fully spatially incoherent monochromatic thermal light source at the fundamental frequency. Whereas the intensity distribution of this light source is often treated as having a smooth intensity profile, it is actually the result of averaging. When the sensing time scale is small enough, it can be viewed as a series of randomly emitting speckle fields, such as atoms undergoing spontaneous emission in the filament of a light bulb. In our work, we aim to synthesize the coherence for the second harmonic that is consistent with the object-induced coherence at the fundamental frequency. This is achieved by first retrieving a series of speckle fields that constitute the fundamental coherence. Subsequently, we digitally impose amplitude and phase modulation on each speckle field. The modulated fields are produced by a spatial light modulator (SLM) at the input plane of the quadratic nonlinear photonic crystal to achieve a second harmonic field with the desired coherence in the far field. Here, the retrieval and modulation of fundamental frequency speckle fields are conducted through numerical simulation, while the synthesis of the second harmonic speckle field is performed experimentally (see Methods). We characterize the speckle field at the object plane as ${{\bf{E}}}_{{\boldsymbol{\omega }}}\left({\bf{r}}\right)={\left[{{\rm{E}}}_{{\boldsymbol{\omega }}}^{1}\left({\bf{r}}\right),\ldots ,{{\rm{E}}}_{{\boldsymbol{\omega }}}^{{\rm{n}}}\left({\bf{r}}\right)\right]}^{\top }$ where ${{\rm{E}}}_{{\boldsymbol{\omega }}}^{{\rm{n}}}\left({\bf{r}}\right)$ represents the n^th^ frame of instantaneous speckle field. Generally, the object-induced coherence (see Methods for the specific case with the smiley face) can be expressed as,

1 $$\begin{array}{l}{{\boldsymbol{\Gamma }}}_{{\boldsymbol{\omega }}}\left({{\bf{r}}}_{1},{{\bf{r}}}_{2}\right)=\left\langle {\left[{{\bf{E}}}_{{\boldsymbol{\omega }}}\left({{\bf{r}}}_{1}\right)\right]}^{* }{\left[{{\bf{E}}}_{{\boldsymbol{\omega }}}\left({{\bf{r}}}_{2}\right)\right]}^{\top }\right\rangle \end{array}$$

where 〈 ⋅ 〉 represents the statistical average over a total of n frames of speckle fields; ${{\bf{r}}}_{{\rm{j}}}={{\rm{x}}}_{{\rm{j}}}\hat{{\bf{x}}}+{{\rm{y}}}_{{\rm{j}}}\hat{{\bf{y}}}$ (or ${{\bf{r}}}_{{\rm{j}}}={{\rm{r}}}_{{\rm{j}}}\hat{{\bf{r}}}+{\uptheta }_{{\rm{j}}}\hat{{{\uptheta }}}$) represents the different transverse coordinates; * and ⊤ represent the complex conjugate and transpose, respectively. Building upon the fundamental frequency coherence, the desired form of the synthesized second harmonic coherence will be,

2 $$\begin{array}{ll}{{\boldsymbol{\Gamma }}}_{2{\boldsymbol{\omega }}}\left({{\bf{r}}}_{1},{{\bf{r}}}_{2}\right)=\left\langle {\left[{{\bf{E}}}_{{\boldsymbol{2\omega }}}\left({{\bf{r}}}_{1}\right)\right]}^{* }{\left[{{\bf{E}}}_{{\boldsymbol{2\omega }}}\left({{\bf{r}}}_{2}\right)\right]}^{\top }\right\rangle \\\qquad\qquad\quad \,=\left\langle {\left[{\hat{{\bf{F}}}}^{-1}\hat{{\bf{N}}}\hat{{\bf{S}}}\hat{{\bf{F}}}{{\bf{E}}}_{{\boldsymbol{\omega }}}\left({{\bf{r}}}_{1}\right)\right]}^{* }{\left[{\hat{{\bf{F}}}}^{-1}\hat{{\bf{N}}}\hat{{\bf{S}}}\hat{{\bf{F}}}{{\bf{E}}}_{{\boldsymbol{\omega }}}\left({{\bf{r}}}_{2}\right)\right]}^{\top }\right\rangle \end{array}$$

where

- $\hat{{\bf{F}}}$ and ${\hat{{\bf{F}}}}^{-1}$ are the Fourier transform operator and its inverse respectively, denoting the far-field propagation based on the fundamental relationship between Fraunhofer diffraction and Fourier transform;
- $\hat{{\bf{N}}}$ is the nonlinear operator that maps the initial fundamental frequency speckles onto the output second harmonic as the speckles go through the nonlinear crystal;
- The operator $\hat{{\bf{S}}}$ denotes a complex field modulation applied to the fundamental frequency speckles, modifying both their amplitude and phase before they interact with the nonlinear crystal.

To demonstrate such a coherence synthesis process in detail, we first numerically retrieve the speckles in the far field $\hat{{\bf{F}}}{{\bf{E}}}_{{\boldsymbol{\omega }}}\left({\bf{r}}\right)$ by capturing the intensity of the speckles at three positions along the optical axis: one in the rear focal plane (${| \hat{{\bf{F}}}{{\bf{E}}}_{{\boldsymbol{\omega }}}\left({\bf{r}},{\rm{z}} = 0\right)| }^{2}$) and the others slightly out of focus (both before and after the focus plane, ${| \hat{{\bf{F}}}{{\bf{E}}}_{{\boldsymbol{\omega }}}\left({\bf{r}},{\rm{z}} = -{{\Delta }}{\rm{z}}\right)| }^{2}$ and ${| \hat{{\bf{F}}}{{\bf{E}}}_{{\boldsymbol{\omega }}}\left({\bf{r}},{\rm{z}} = {{\Delta }}{\rm{z}}\right)| }^{2}$) by a defocus distance Δz. The intensities collected at these three planes are utilized in a phase unwrapping algorithm^25,26^ that returns the complex valued expression for each instantaneous far-field speckle. In particular, the phase of the retrieved speckle field by the algorithm is represented as a continuous phase rather than a wrapped phase, which ensures that no global information will be lost in subsequent modulation processes. Once we have gathered all the information about the speckle field that establishes the coherence of the far-field object in each frame, we apply a complex field modulation to the speckle which is denoted as $\hat{{\bf{S}}}\hat{{\bf{F}}}{{\bf{E}}}_{{\boldsymbol{\omega }}}\left({\bf{r}}\right)$. The design of this modulator $\hat{{\bf{S}}}$ is critical. To properly configure it, it is essential to first comprehend the effects on the speckles as they undergo the nonlinear process. As shown in Fig. 2, the experimental setup (see Methods for details) essentially performs as a nonlinear self-imaging system that consists of an SLM and a 4*f* configuration with a quadratic nonlinear photonic crystal arranged in the Fourier space. When the laser passes through the SLM, it generates the modulated speckle fields $\hat{{\bf{S}}}\hat{{\bf{F}}}{{\bf{E}}}_{{\boldsymbol{\omega }}}\left({\bf{r}}\right)$ at the incident plane of the nonlinear crystal in this system’s spatial frequency domain. They subsequently result in the second harmonic speckles $\hat{{\bf{N}}}\hat{{\bf{S}}}\hat{{\bf{F}}}{{\bf{E}}}_{{\boldsymbol{\omega }}}\left({\bf{r}}\right)$ at the output plane of the crystal. Consequently, we synthesize the desired second harmonic speckle fields ${\hat{{\bf{F}}}}^{-1}\hat{{\bf{N}}}\hat{{\bf{S}}}\hat{{\bf{F}}}{{\bf{E}}}_{{\boldsymbol{\omega }}}\left({\bf{r}}\right)$ in the spatial domain of the nonlinear self-imaging system upon an inverse Fourier transform.

From the comparison between Eqs. (1), (2), it is evident that synthesizing a second harmonic with the same coherence as the fundamental light requires that the combination of the nonlinear operator $\left(\hat{{\bf{N}}}\right)$ and the modulation operator $\left(\hat{{\bf{S}}}\right)$ results in the identity operation, meaning $\hat{{\bf{S}}}$ equals the inverse of $\hat{{\bf{N}}}\,\left(\hat{{\bf{S}}}={\hat{{\bf{N}}}}^{-1}\right)$. Determining the corresponding inverse relationship between two such operators is often challenging, as the nonlinear coupled wave equations involved typically lack an analytical solution. However, under the weak interaction approximation, the act of the nonlinear operator on the fundamental frequency light can be expressed as proportional to the square of the fundamental frequency light’s complex field, i.e., $\hat{{\bf{N}}}{\bf{E}}\left({\bf{r}}\right)\propto {\left[{\bf{E}}\left({\bf{r}}\right)\right]}^{2}$ (see Supplementary Note 1). In practice, such an approximation is achievable experimentally using a nonlinear crystal having a much shorter length than the confocal parameters of the interacting beams. To counteract the spatial nonlinear effect in our experiment shown in Fig. 2, we therefore set the modulation of the recovered far-field fundamental frequency speckle field as the square root of its field, that is, $\hat{{\bf{S}}}{\bf{E}}\left({\bf{r}}\right)=\sqrt{{\bf{E}}\left({\bf{r}}\right)}$. For more general cases where strong nonlinear interactions are triggered, $\hat{{\bf{N}}}{\bf{E}}\left({\bf{r}}\right)$ can be computed using a numerical solver—such as the split-step Fourier method^27^—acting on the fundamental field at the crystal’s input plane. $\hat{{\bf{S}}}{\bf{E}}\left({\bf{r}}\right)$, on the other hand, could be determined using a learning-based approach, with the model trained to approximate the inverse by minimizing the discrepancy between the coherence functions $\langle {[\hat{{\bf{N}}}\hat{{\bf{S}}}{{\bf{E}}}_{{\boldsymbol{\omega }}}({{\bf{r}}}_{1})]}^{* }{[\hat{{\bf{N}}}\hat{{\bf{S}}}{{\bf{E}}}_{{\boldsymbol{\omega }}}({{\bf{r}}}_{2})]}^{\top }\rangle$ and $\left\langle {\left[{{\bf{E}}}_{{\boldsymbol{\omega }}}\left({{\bf{r}}}_{1}\right)\right]}^{* }{\left[{{\bf{E}}}_{{\boldsymbol{\omega }}}\left({{\bf{r}}}_{2}\right)\right]}^{\top }\right\rangle$. But in this work, we focus specifically on implementing the weak interaction approximation.

**Fig. 3:**
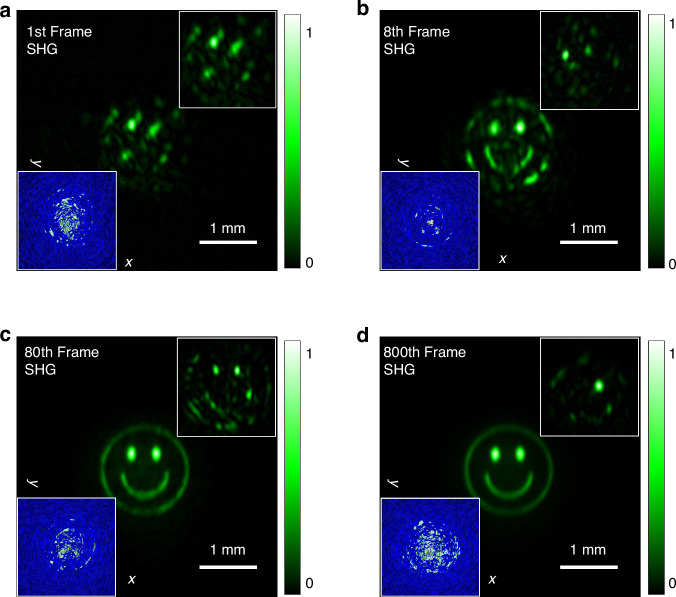
Multi-frame holograms and synthesized speckle patterns depicting second harmonic coherence. **a–d** Statistical averages of speckle intensities shown for increasing frame ranges: frame 1, frames 1-8, frames 1-80, and frames 1-800. The insets at the lower left corner show the 1**st**, 8**th**, 80**th**, and 800**th** frames in the hologram movie on the SLM screen, respectively. Correspondingly, the insets at the upper right corner display the synthesized individual speckle intensities. Both sets of intensity images are presented on a consistent scale. The hologram patterns are composed of 640 × 640 pixels

The smiley face is illuminated by a monochromatic thermal source, realized by imprinting the desired phase pattern at the fundamental frequency using the SLM. This approach facilitates convenient control over the coherence properties of the light source (see Methods). We retrieve 800 frames of speckle fields, $\left[\hat{{\bf{F}}}{{\rm{E}}}_{{\boldsymbol{\omega }}}^{1}\left({\bf{r}}\right),\ldots ,\hat{{\bf{F}}}{{\rm{E}}}_{{\boldsymbol{\omega }}}^{800}\left({\bf{r}}\right)\right]$, that constitute the far-field coherence of the smiley face. The inset in Fig. 2 illustrates the true continue phase $\varphi \left({\rm{x}},{\rm{y}}\right)$ of the speckle fields for the 1^**st**^, 8^**th**^, 80^**th**^, and 800^**th**^ frames, each with a size of 1080 × 1080 pixels. It can be seen that the phase value of each pixel randomly varies between the range of $\left[-5\pi ,5\pi \right]$. After applying the square root modulation, the resultant phase $\phi \left({\rm{x}},{\rm{y}}\right)$ of the speckle fields, shown in the subsequent inset in Fig. 2, is halved from $\varphi \left({\rm{x}},{\rm{y}}\right)$ and wrapped within the range of $\left[-\uppi ,\uppi \right]$, defined as $\phi \left({\rm{x}},{\rm{y}}\right)={\bf{arg}}\left[\exp \left({\rm{i}}\varphi \left({\rm{x}},{\rm{y}}\right)/2\right)\right]$. This wrapping, essential for hologram processing, leads to the sawtooth-shaped phase distribution. Subsequently, each frame of square-root-modulated complex field information is processed into a phase-only hologram^28^ and displayed on the SLM. As depicted in the experimental setup in Fig. 2, a fully coherent laser operating at the fundamental frequency illuminates the SLM and is directed into a nonlinear crystal. The synthesized second harmonic speckles are then measured in the crystal’s far field. A sequence of 800 holograms was played on the SLM as a movie at a refresh rate of 60 Hz. The insets at the lower left corner of Fig. 3a-d show the 1^**st**^, 8^**th**^, 80^**th**^, and 800^**th**^ frames in the hologram sequence, respectively. Correspondingly, we synthesized 800 frames of second harmonic speckles. Figure 3a-d illustrate the cumulative results from these frames, with each independently synthesized speckle compared in the upper right corner. For instance, Fig. 3d presents the statistical average of the intensity from the 1^**st**^ to the 800^**th**^ frame, while the upper right corner distinctly shows the speckle intensity of the 800^**th**^ frame. It can be seen that although each individual speckle is random, the overall intensity of these speckles gradually forms a smiley face through superposition (see Supplementary Video 1 for the detailed experimental formation process). These results confirm that the coherence ${{\boldsymbol{\Gamma }}}_{{\boldsymbol{2\omega }}}\left({{\bf{r}}}_{1},{{\bf{r}}}_{2}\right)$ of the second harmonic, composed of these speckles, accurately replicates the coherence ${{\boldsymbol{\Gamma }}}_{{\boldsymbol{\omega }}}\left({{\bf{r}}}_{1},{{\bf{r}}}_{2}\right)$ of the fundamental frequency light induced by the initial object.

### Nonlinear generation of incoherent vortex beams

Next, we apply this coherence synthesis method to the nonlinear generation of incoherent structured light beams. Initially, we study the incoherent vortex beams^29^ characterized by Laguerre-Gaussian (LG) modes. We denote the speckle fields of a desired second harmonic incoherent vortex beam at the crystal’s output plane as

3 $$\begin{array}{l}{{\bf{E}}}_{{\boldsymbol{2\omega }}}^{{\rm{o}}}\left({\bf{r}}\right)={{\rm{LG}}}_{p}^{\ell }\left({\bf{r}}\right)\exp \left[-{\rm{i}}{{\boldsymbol{\phi }}}_{r}\left({\bf{r}}\right)\right]\end{array}$$

where ${{\rm{LG}}}_{p}^{\ell }\left({\bf{r}}\right)$ is the LG mode expression—an exact solution to the paraxial Helmholtz equation^30^ with the orbital angular momentum (OAM) index *ℓ* that measures the OAM of each photon in the units of *ℏ* and the radial index *p*; The phase collections ${{\boldsymbol{\phi }}}_{{\rm{r}}}\left({\bf{r}}\right)={\left[{\phi }_{{\rm{r}}}^{1}\left({\bf{r}}\right),\ldots ,{\phi }_{{\rm{r}}}^{{\rm{n}}}\left({\bf{r}}\right)\right]}^{\top }$ with the n^th^ frame of instantaneous phase are stochastic, influencing the correlation of speckle fields that comprise the incoherent vortex beams. For simplicity and without loss of generality, we focus on pure OAM modes by setting the radial index *p* to zero. At the same time, we adjust the random phase to follow Gaussian statistics. The coherence function for the second harmonic incoherent vortex beams thus takes the form as

4 $$\begin{array}{ll}{{\boldsymbol{\Gamma }}}_{2{\boldsymbol{\omega }}}^{{\rm{o}}}\left({{\bf{r}}}_{1},{{\bf{r}}}_{2}\right)\,=\left\langle {\left[{{\bf{E}}}_{{\boldsymbol{2\omega }}}^{{\rm{o}}}\left({{\bf{r}}}_{1}\right)\right]}^{* }{\left[{{\bf{E}}}_{{\boldsymbol{2\omega }}}^{{\rm{o}}}\left({{\bf{r}}}_{2}\right)\right]}^{\top }\right\rangle \\\qquad\qquad\qquad \,={\left(\frac{{{\bf{r}}}_{1}{{\bf{r}}}_{2}}{{{\rm{w}}}_{0}}\right)}^{\ell }\exp \left[-\frac{{{\rm{r}}}_{1}^{2}+{{\rm{r}}}_{2}^{2}}{2{{\rm{w}}}_{0}^{2}}-{\rm{i}}\ell ({\theta }_{1}-{\theta }_{2})-\frac{{\left({{\bf{r}}}_{1}-{{\bf{r}}}_{2}\right)}^{2}}{2{\sigma }_{\mu }^{2}}\right]\end{array}$$

where w_0_ and *σ*_*μ*_ denote the beam width and the coherence width respectively. Considering the relationship between the incident fundamental frequency field and the output second harmonic field, it becomes clear that the required fundamental frequency speckle field once again represents an incoherent vortex beam. In Supplementary Note 2, we demonstrate that the random phase correlation of the fundamental frequency field also follows a Gaussian distribution, with a coherence width that is twice that of the second harmonic. This is crucial for constructing a fundamental frequency pump source to accurately achieve the desired coherence in the second harmonic. In particular, the topological charge of the synthesized incoherent vortex beam will be twice that of the fundamental frequency light, i.e.,

5 $${\ell }_{2\omega }=2{\ell }_{\omega }$$

This conservation law assumes that the crystal does not contribute orbital angular momentum to the process. But if the crystal is properly structured, its contribution must also be taken into account^31,32^.

**Fig. 4:**
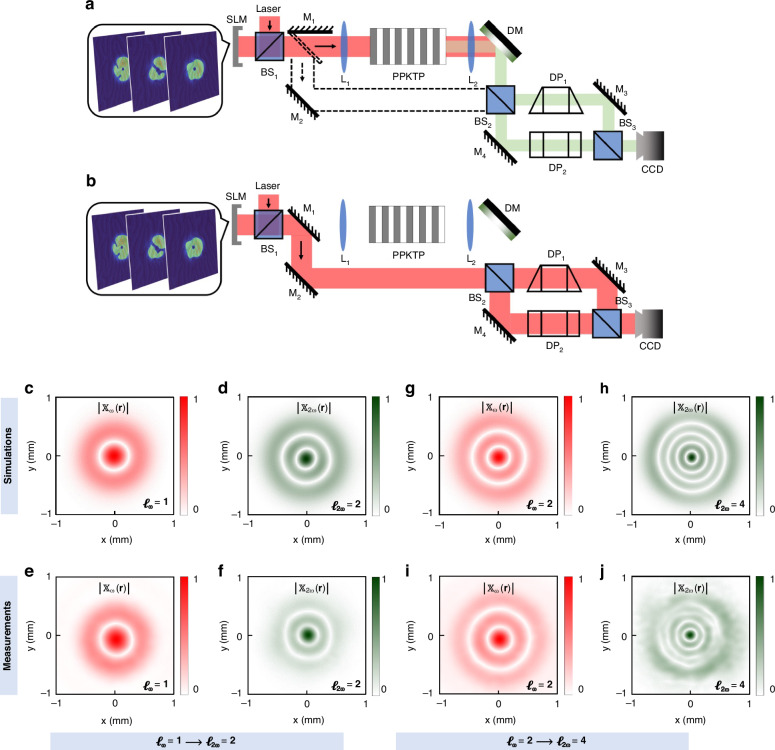
Overview of the experimental setups and their functions. **a** A schematic depicting the apparatus for nonlinearly synthesizing the second harmonic incoherent vortex beams and measuring their far-field cross correlation function to determine the topological charges. **b** Examining the topological charges of the fundamental frequency pump beams. **c–f** Numerical simulations and experimental measurements of the far-field cross correlation function for the case where the second harmonic incoherent vortex beams (green) with *ℓ*_2*ω*_ = 2 are synthesized from the fundamental frequency counterparts (red) with *ℓ*_*ω*_ = 1. **g–j** The case where the beams with *ℓ*_2*ω*_ = 4 are synthesized from those with *ℓ*_*ω*_ = 2. Other parameters are *σ*_*μ*_ = w_0_ = 100 μm. SLM, spatial light modulator; M_1_, M_2_, M_3_, and M_4_, mirrors; L_1_ and L_2_, lenses; BS_1_, BS_2_, and BS_3_, beam splitters; PPKTP, periodically poled KTiOPO_4_ nonlinear crystal; DM, dichroic mirror; DP_1_ and DP_2_, dove prisms; CCD, charge-coupled device

In this experiment, we continue to utilize the nonlinear self-imaging system as previously described. Moreover, to observe the synthesis process of the incoherent vortex beam, we utilize a type of Mach-Zehnder interferometer configured as the wavefront folding interferometer^29^, with each arm containing a Dove prism oriented in opposite directions. To achieve precise interferometric conditions, we ensure that the intensity level of the speckle patterns remains identical in each arm. These setups depicted in Fig. 4a,b enable the measurements of the far-field cross-correlation functions of both the second harmonic and fundamental frequency light, ${{\mathbb{X}}}_{2\omega }\left({\bf{r}}\right)={{\boldsymbol{\Gamma }}}_{2\omega }\left({\bf{r}},-{\bf{r}}\right)$ and ${{\mathbb{X}}}_{\omega }\left({\bf{r}}\right)={{\boldsymbol{\Gamma }}}_{\omega }\left({\bf{r}},-{\bf{r}}\right)$, respectively. We can determine the number of topological charges by simply counting the number of ring singularities from the measured interference patterns. Figure 4c-j present the numerical simulations and experimental measurements of the cross-correlation functions for the fundamental frequency beams (red) and the second harmonic generation (green). The left panel (see Fig. 4c–f) shows that the fundamental frequency beams with the single ring singularity in their spatial coherence (see Fig. 4c, e), after undergoing nonlinear interactions with the *χ*^(2)^ crytals, result in the double ring singularities in the second harmonic beams (see Fig. 4d, f). The observed doubling of ring singularities in the second harmonic beams not only implies that the nonlinear interactions efficiently transfer and enhance the topological charge associated with the input fundamental frequency beams, but also indicates that the second harmonic incoherent vortex beams with *ℓ*_2*ω*_ = 2 are nonlinearly synthesized by the fundamental frequency counterparts with *ℓ*_*ω*_ = 1. More importantly, since the incoherent vortices are based on the LG eigenmodes carrying an OAM of *ℓℏ* per photon, these results unveil the fact that the conservation law for the OAM of light in the nonlinear interactions remains valid even under the partially incoherent state. Moreover, the *ℓ*_*ω*_ = 2-to-*ℓ*_2*ω*_ = 4 case is also studied. As shown in the right panel (see Fig. 4g–j), the four ring singularities observed in the far-field cross-correlation function of the second harmonic beams (see Fig. 4h, j) are produced by pumping the fundamental frequency beams which themselves exhibit two ring singularities in their far-field cross-correlation function (see Fig. 4g, i). The presence of these doubled singularities shown in the measured coherence function confirms the coherence synthesis and transformation of incoherent optical vortices through nonlinear frequency conversion, upon the OAM conservation law. Please note that although the synthesis of the incoherent vortex beam in this work relies on a laser source and a hologram of LG modes with a random phase distribution, it is also possible to achieve similar results using an incoherent light source—such as an incandescent lamp—together with a spiral phase plate designed for the corresponding wavelength. In Supplementary Videos 2 and 3, we show the dynamic measurements in detail, illustrating the evolution from individual frames of the interference patterns to the post-processed far-field cross-correlation function.

### Nonlinear generation of incoherent Airy beams

Having explored the capabilities of nonlinearly synthesizing incoherent vortex beams, particularly their ability to impart OAM in nonlinear interactions, we now turn our attention to Airy beams^33–35^. Unlike vortex beams, Airy beams offer unique “non-diffracting” and “self-accelerating” properties, making them ideal for applications requiring precise trajectory control over long distances. It has traditionally been assumed that the formation of Airy beams, whether in linear^34^ or nonlinear optics^36^, necessitates the use of fully coherent light sources, as the stability and persistence of these unique properties are contingent upon a well-defined phase relationship across the wavefront. Recent studies in linear optics^37,38^ have demonstrated that low-coherence light can also achieve shape-preserving acceleration similar to that of fully coherent beams, provided that the electric dipoles from the incoherent sources collectively exhibit coherent superposition with each accelerating component. In the realm of nonlinear optics, however, generating an incoherent Airy beam that exhibits acceleration over a long interaction length, comparable to its fully coherent counterpart, remains a significant challenge.

For this purpose, we expect the coherence function at the crystal’s output plane to be

6 $$\begin{array}{l}{{\boldsymbol{\Gamma }}}_{2{\boldsymbol{\omega }}}^{{\rm{o}}}\left({{\bf{r}}}_{1},{{\bf{r}}}_{2}\right)=\exp \left[-\frac{{{\bf{r}}}_{1}^{2}+{{\bf{r}}}_{2}^{2}}{2{{\rm{w}}}_{0}^{2}}-\frac{{\rm{i}}}{3}{\gamma }_{2\omega }^{3}\left({{\bf{r}}}_{1}^{3}-{{\bf{r}}}_{2}^{3}\right)-\frac{{\left({{\bf{r}}}_{1}-{{\bf{r}}}_{2}\right)}^{2}}{2{\sigma }_{\mu }^{2}}\right]\end{array}$$

where *γ*_2*ω*_ is the modulation scale of the cubic phase. Here we use the Fourier transform relations between the Airy function and a cubic phase function in the Fourier space. At the far field of the crystal’s output plane, we can utilize the nonlinear filtering model (see Supplementary Note 3) to write down the coherence function as

7 $$\begin{array}{ll}{{\boldsymbol{\Gamma }}}_{2{\boldsymbol{\omega }}}\left({{\bf{r}}}_{1},{{\bf{r}}}_{2},{\rm{z}}\right)\,={\iint }_{-\infty }^{\infty }{{\boldsymbol{\Gamma }}}_{2{\boldsymbol{\omega }}}^{{\rm{o}}}\left({{\bf{r}}}_{1}^{{\prime} },{{\bf{r}}}_{2}^{{\prime} }\right)\exp \left[-\frac{{\rm{i}}{{\rm{k}}}_{2}\left({{\bf{r}}}_{2}\cdot {{\bf{r}}}_{2}^{{\prime} }-{{\bf{r}}}_{1}\cdot {{\bf{r}}}_{1}^{{\prime} }\right)}{{\rm{f}}}\right]\,{d}^{2}{{\bf{r}}}_{1}^{{\prime} }\,{d}^{2}{{\bf{r}}}_{2}^{{\prime} },\\\qquad\qquad\qquad \,=\sum _{{\rm{n}}}{\alpha }_{{\rm{n}}}\left(\delta \right){\uppsi }^{* }\left({{\bf{r}}}_{1},{\rm{z}}\right)\uppsi \left({{\bf{r}}}_{2},{\rm{z}}\right)\end{array}$$

with

8 $$\begin{array}{l}\uppsi \left({\bf{r}},{\rm{z}}\right)={\rm{Ai}}\left(\frac{{\bf{r}}-\delta }{{{\rm{r}}}_{0}}-\frac{{{\rm{z}}}^{2}}{4{{\rm{z}}}_{0}^{2}}+{\rm{i}}{\rm{a}}\frac{z}{{{\rm{z}}}_{0}}\right)\exp \left({\rm{a}}\frac{{\bf{r}}-\delta }{{{\rm{r}}}_{0}}-{\rm{a}}\frac{{{\rm{z}}}^{2}}{2{{\rm{z}}}_{0}^{2}}-{\rm{i}}\frac{{{\rm{z}}}^{3}}{12{{\rm{z}}}_{0}^{3}}+{\rm{i}}\frac{{{\rm{a}}}^{2}{\rm{z}}}{2}+{\rm{i}}\frac{\left({\bf{r}}-\delta \right){\rm{z}}}{2{{\rm{r}}}_{0}{{\rm{z}}}_{0}}\right)\end{array}$$

where ${\rm{Ai}}\left(\cdot \right)$ is the Airy function^39^; ${\alpha }_{{\rm{n}}}\left(\delta \right)$ is the random weight function about the transverse displacement *δ*; a is the decay factor; f is the focal length of the Fourier lens; r_0_ = *γ*_2*ω*_f/k_2_ is the characteristic transverse width of each coherent second harmonic Airy mode; ${{\rm{z}}}_{0}={{\rm{k}}}_{2}{{\rm{r}}}_{0}^{2}$ is the Rayleigh distance. We can see from Eq. (7) the synthesized coherence function consists of a series of transversely shifted Airy modes, all having the same acceleration coefficient. The parabolic acceleration for the second harmonic is determined by the arguments inside the Airy function as

9 $$\begin{array}{l}\frac{{\bf{r}}-\delta }{{{\rm{r}}}_{0}}-\frac{{{\rm{z}}}^{2}}{4{{\rm{z}}}_{0}^{2}}=0\to {\bf{r}}={\beta }_{2\omega }{{\rm{z}}}^{2}\end{array}$$

with the acceleration factor ${\beta }_{2\omega }={{\rm{k}}}_{2}/4{\gamma }_{2\omega }^{3}{{\rm{f}}}^{3}$. The coherence function of the fundamental frequency beams incident to the crystal exhibits a structural similarity to Eq. (6). Both functions represent the scene where a Gaussian Schell-model light source is modulated by a cubic phase. However, the modulation scales of the two beams differ. Specifically, the modulation scale *γ*_*ω*_ at the fundamental frequency compared to the one *γ*_2*ω*_ of the synthesized second harmonic is given by the ratio *γ*_*ω*_/*γ*_2*ω*_ = 2^−1/3^. More importantly, the acceleration factor for the fundamental frequency beams is identical to that for the second harmonic, i.e.,

10 $${\beta }_{\omega }=\frac{{{\rm{k}}}_{1}}{4{\gamma }_{\omega }^{3}{{\rm{f}}}^{3}}=\frac{{{\rm{k}}}_{2}/2}{4{\left({2}^{-1/3}{\gamma }_{2\omega }\right)}^{3}{{\rm{f}}}^{3}}=\frac{{{\rm{k}}}_{2}}{4{\gamma }_{2\omega }^{3}{{\rm{f}}}^{3}}={\beta }_{2\omega }$$

**Fig. 5:**
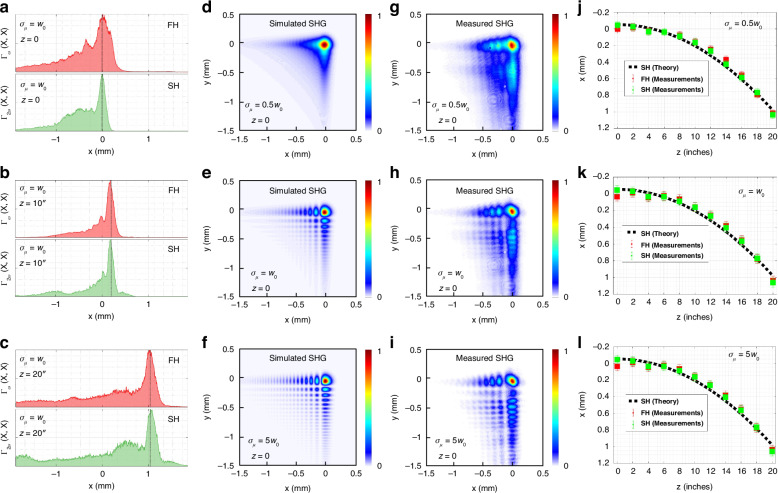
Numerical simulations and experimental results. **a–c** Measurements of one-dimensional fundamental frequency and second harmonic incoherent Airy beams with the coherence width *σ*_*μ*_ = w_0_ for various propagation distances *z* = 1, 10, 20 inches. **d–i** Intensity of two-dimensional synthesized second harmonic incoherent Airy beams at the initial plane *z* = 0 for various coherence width *σ*_*μ*_ = 0.5w_0_, w_0_, 5w_0_. **j–l** Dynamic evolution of peak intensity for fundamental frequency and second harmonic incoherent Airy beams along the propagation axis. Here, the beam width is set as w_0_ = 100 μm

The identical acceleration of the fundamental frequency and second harmonic Airy beams was observed previously for fully coherent beams^40,41^. As we show here, this feature also holds for second harmonic generation of incoherent beams. For simplicity, we first focus on the one-dimensional case. Figure 5a-c present the measured intensity ${{\boldsymbol{\Gamma }}}_{{\boldsymbol{\omega }}}\left({\rm{x}},{\rm{x}}\right)$ of an incoherent Airy beam at the fundamental frequency, alongside the intensity ${{\boldsymbol{\Gamma }}}_{{\boldsymbol{2\omega }}}\left({\rm{x}},{\rm{x}}\right)$ of a synthesized incoherent Airy beam at the second harmonic, observed at various propagation distances. We can see that over the propagation from z = 1 inch to z = 20 inches, the peaks of both the fundamental frequency and the second harmonic incoherent Airy beam with the coherence width *σ*_*μ*_ = 0.5w_0_, as denoted by the gray dotted lines, maintain synchronization throughout the propagation. This observation indicates that both beams exhibit the joint acceleration characteristics. Further explorations into the influence of the coherence width on these incoherent Airy beams are presented in Fig. 5d-f, which detail the simulation results for the two-dimensional synthesized incoherent Airy beam with various coherence width at the initial plane. They reveal that as the coherence diminishes, the side lobes of the beam progressively vanish, while the energy in the main lobe becomes increasingly focused. The experimental validations of these simulations are illustrated in Fig. 5g–i (see Supplementary Video 4 for each frame of synthesized second harmonic incoherent Airy beam). Moreover, Fig. 5j–l illustrate the evolution in peak intensities of both the fundamental frequency and second harmonic incoherent Airy beams over the propagation distance with the coherence width *σ*_*μ*_ = 0.5w_0_, w_0_, and 5w_0_, respectively. These observations confirm that all configurations share the same acceleration curve, thereby indicating that coherence does not affect the acceleration of the synthesized second harmonic incoherent Airy beam.

## Discussion

In conclusion, we have proposed and experimentally validated a method for the synthesis of spatial coherence in nonlinear optical interactions. Our approach for nonlinearly synthesizing object-induced coherence involves first reconstructing the fundamental frequency speckle pattern for each frame, followed by the square root filtering to counteract the square modulation imposed by the nonlinear crystal on the incident light field under weak interactions. This methodology is implemented within our proposed nonlinear self-imaging system. Additionally, this system has been utilized to nonlinearly synthesize incoherent structured light beams. We successfully generated the second harmonic incoherent vortex beams with OAM of 2*ℏ* and 4*ℏ* using fundamental frequency incoherent vortex beams carrying OAM of 1*ℏ* and 2*ℏ* respectively, thereby demonstrating the conservation of OAM under low coherence conditions. Moreover, incoherent self-accelerating Airy beams were also synthesized in the second harmonic. Notably, the incoherent Airy beams generated by our method exhibit identical acceleration distances and rates to those of their fully coherent modes, irrespective of coherence width. In this study, we have considered the scenario of an incoherent pump interacting with an ordered nonlinear crystal. On the other hand, the principles discussed are also applicable to cases involving a coherent pump and randomly structured nonlinear crystals^42–45^. In order to average over many various random realizations, the crystal itself can be either shifted or rotated, so that each time the pump passes through a different part of the crystal.

Object-induced coherence synthesis satisfies the need for the transfer of coherence from infrared to visible light frequencies, offering further understanding for the role of incoherent light in infrared imaging processes. The nonlinearly synthesized incoherent Airy beams are anticipated to enhance the performance of light-sheet microscopy^46^, as the suppression of side lobes—achieved through low coherence—could reduce unwanted fluorescence outside the region of interest. In addition, since these beams are generated through nonlinear frequency conversion, the findings may facilitate more precise excitation of fluorophores at their optimal wavelengths. Although our results establish a fundamental proof-of-concept for synthesizing optical spatial coherence in nonlinear frequency conversion, several intriguing questions about the scalability of our method remain open, including: Can the proposed synthesis method be realized in cases of phase mismatch due to environmental factors? If so, what role would the phase-matching condition play in the nonlinear interaction of incoherent light? Could this method be scaled to higher-order nonlinear media, enabling phenomena such as third harmonic generation or four wave mixing? Further developments should also address practical issues such as laser stability, crystal imperfections, and environmental fluctuations, which could degrade coherence and impact the method’s long-term reliability. Beyond the degree of freedom of spatial coherence, our method may generalize to nonlinear control of temporal coherence simultaneously. For instance, the implementations of nonlinear crystals encoded with spectral holograms, combined with the incoherent amplified spontaneous emission of an erbium-doped fiber amplifier as a light source^47^, could enable full-scale spatiotemporal coherence control. We believe our method presented in this article might pave the way towards nonlinear interactions of incoherent light and their applications in infrared imaging and biosensing.

## Methods

### Thermal light source preparation and object-induced coherence

In this section, we detail the numerical creation of a monochromatic thermal source with adjustable spatial coherence, used as the fundamental frequency illumination source for the object in Fig. 1. We first model a scattering scenario where a fully coherent laser source with a Gaussian envelope illuminates a diffuser characterized by a random phase plate positioned at the beam waist. As the laser interacts with the diffuser, it generates speckles due to constructive and destructive interference across various spatial regions, resulting in a fully spatially coherent pattern^48^. To reduce coherence, we simulate multiple realizations by continuously updating the random phase on the SLM, thereby averaging the fluctuations from each scattering event. This process mimics pseudo-thermal light, achieving a coherence comparable to that of an actual thermal source^49^. The stationary pseudo-thermal light source operates at a power of 1 mW, with a spectral profile characterized by a narrow bandwidth centered at a wavelength of 1064 nm. We create the illumination source with a spatial coherence area 100 times smaller than the object. Specifically, the smiley face image shown in Fig. 3 has a dimension of approximately 1 × 1 mm^2^, while the light source’s coherence area is thus set as 100 × 100 μm^2^. This discrepancy in coherence area renders the source effectively incoherent. We carried out simulations of 800 scattering events, where each resulting speckle pattern served as the illumination for the smiley face. When added together, the coherence function on the object plane at the fundamental frequency is derived as,11with the smiley face shown in the two different transverse coordinates **r**_1_ and **r**_2_. Based on this expression, we verify the synthesis of four-dimensional coherence function for the second harmonic by measuring the intensity ${{\boldsymbol{\Gamma }}}_{{\boldsymbol{2\omega }}}\left({\bf{r}},{\bf{r}}\right)={{\boldsymbol{\Gamma }}}_{{\boldsymbol{\omega }}}\left({\bf{r}},{\bf{r}}\right)$ which is the two-dimensional projection of Eq. (11) with **r** = **r**_1_ = **r**_2_.

### Experimental setup

The nonlinear synthesis process of object-induced coherence and structured light beam’s coherence shares an experimental setup, as shown in Figs. 2, 4. A collimated, linearly polarized Nd:YAG Q-switched laser beam (centered at *λ* = 1064.5 nm with a bandwidth of less than 0.6 nm, pulse energy of 50 μJ, pulse width of 1 ns, and repetition rate of 1 kHz) illuminates the SLM (HoloEye PLUTO-2.1-NIR-133 with 1920 × 1080 pixels of pitch 8 *μ*m and calibration for a 2*π* phase shift at *λ* = 1064.5 nm). The laser operates at an average power of 50 mW and achieves a peak power of 50 kW. On the SLM screen, we have encoded a sequence of holograms designed to continuously generate 800 frames of speckle fields, which collectively form the desired coherence. For object-induced coherence, the hologram sequence represents the fields $\left[\hat{{\bf{F}}}\hat{{\bf{S}}}\hat{{\bf{F}}}{{\rm{E}}}_{{\boldsymbol{\omega }}}^{1}\left({\bf{r}}\right),\ldots ,\hat{{\bf{F}}}\hat{{\bf{S}}}\hat{{\bf{F}}}{{\rm{E}}}_{{\boldsymbol{\omega }}}^{800}\left({\bf{r}}\right)\right]$, while for the coherence of the structured beams, the fields are represented by the sequence $\left[\hat{{\bf{F}}}{{\rm{E}}}_{{\boldsymbol{2\omega }}}^{{\rm{o}},1}\left({\bf{r}}\right),\ldots ,\hat{{\bf{F}}}{{\rm{E}}}_{{\boldsymbol{2\omega }}}^{{\rm{o}},800}\left({\bf{r}}\right)\right]$. The additional Fourier transform operator $\hat{{\bf{F}}}$ arises from the phase-only hologram algorithm^28^. When a fully coherent laser beam interacts with the SLM encoded with these holograms and is subsequently focused by a thin lens, the partially coherent pump beam with the desired coherence is accurately created onto the crystal’s incident plane upon an inverse Fourier transform. Throughout our work, we use a 2-mm-long KTiOPO_4_ nonlinear crystal periodically poled in the *z* direction with the period of 9 *μ*m. The crystal temperature is tuned to 32 ^∘^C to satisfy collinear quasi-phase matching for second harmonic at 532 nm. While this work employs quasi-phase matching, it can alternatively be implemented using a birefringent phase matching scheme with a Beta-Barium Borate crystal, which eliminates the need for a temperature-controlled environment and simplifies the experimental setup. To the end, we achieve the second harmonic field with the desired coherence in the far field.

## Supplementary information

Supplementary information for Coherence synthesis in nonlinear optics

Nonlinear synthesis of object-induced coherence.

Nonlinear generation of incoherent vortex beams from l_ω ℏ=1ℏ to l_2ω ℏ=2ℏ

Nonlinear generation of incoherent vortex beams from l_ω ℏ=2ℏ to l_2ω ℏ=4ℏ

Nonlinear generation of incoherent Airy beams.

## Data Availability

All data used in this study are available from the corresponding authors upon reasonable request.

## Code availability

All custom codes used in this study are available from the corresponding authors upon reasonable request.
