# Supplementary material for: Coherence synthesis in nonlinear optics
Source: Light Sci Appl. 2025 Feb 26;14:101. doi: 10.1038/s41377-025-01749-6 (PMC11862224; doi:10.1038/s41377-025-01749-6)
Supplement: Supplementary file 1 — Supplementary information for Coherence synthesis in nonlinear optics [file 41377_2025_1749_MOESM1_ESM.docx]

Supplementary information for

**Coherence synthesis in nonlinear optics**

Zihao Pang* and Ady Arie^†^

School of Electrical Engineering,

Iby and Aladar Fleischman Faculty of Engineering,

Tel Aviv University, Tel Aviv 69978, Israel

*Corresponding author. Email: [its.zihaopang@gmail.com](mailto:its.zihaopang@gmail.com)

^†^Corresponding author. Email: ady@tauex.tau.ac.il

**This PDF file includes:**

Supplementary Text

Fig. S1

References (1 to 5) (these refer only to references in the Supplementary information)

**Other Supplementary information for this manuscript include the following:**

Videos 1 to 4

Supplementary Text

Supplementary Note 1: Analytical solutions for nonlinear coupled wave equation under the weak interaction approximation

In this section, we build up the theoretical model that captures the nonlinear processes in the interaction. Under the undepleted pump approximation, and assuming slowly varying fields and negligible diffraction, the evolution of the second harmonic beam $E_{2\omega}$ in a quadratic nonlinear photonic crystal can be formulated as the following equation [1],

|  | $\frac{dE_{2\omega}\left( \mathbf{r},z \right)}{\mathrm{dz}}=\frac{i\kappa}{2k_{2}}E_{\omega}^{2}\left( \mathbf{r},z \right)e^{i\Delta kz},$ | (S1) |
| --- | --- | --- |

where $\mathbf{r=}x\hat{\mathbf{x}}+y\hat{\mathbf{y}}$ is the transverse coordinate; $\Delta k=2k_{1}-k_{2}$ is the phase mismatch; $k_{1,2}$ are the wave numbers of the fundamental frequency and second harmonic waves respectively; $\kappa=4{\chi^{(2)}\omega^{2}}/{c^{2}}$ is the nonlinear coupling coefficient; $c$ is the speed of light in vacuum; $\omega$ is the frequency; $\chi^{(2)}$ is the second order susceptibility.

Throughout the work, the nonlinear process is quasi-phase matched so that the term $e^{i\Delta kz}$ is canceled. The solution to Eq. S1 is

|  | $E_{2\omega}\left( \mathbf{r},L \right)\approx\frac{i\kappa L}{2k_{2}}E_{\omega}^{2}\left( \mathbf{r},0 \right),$ | (S2) |
| --- | --- | --- |

where $L$ is the length of the crystal. Consequently, given the fundamental frequency field $E_{\omega}\left( \mathbf{r},0 \right)$ at the incident plane, the output second harmonic field $E_{2\omega}\left( \mathbf{r},L \right)$ can be directly determined using Eq. S2. Conversely, to synthesize a desired $E_{2\omega}\left( \mathbf{r},L \right)$, we can apply a square root operation to obtain the required fundamental frequency field $E_{\omega}\left( \mathbf{r},0 \right)$. It is crucial to consider the phase: the phase of the synthesized second harmonic will be twice that of the fundamental frequency light field.

Supplementary Note 2: Gaussian-correlated structured beams in nonlinear interactions

Here, we will derive the relationship between the coherence of the fundamental frequency light and the second harmonic when the incoherent structured light exhibits Gaussian correlation. The speckle fields of an incoherent structured light beam at the fundamental frequency can be represented as

|  | $E_{\omega}\left( \mathbf{r} \right)=E_{\omega}^{\mathrm{co}}\left( \mathbf{r} \right)\exp\left[ -i\Phi_{\omega}\left( \mathbf{r} \right) \right].$ | (S3) |
| --- | --- | --- |

When these fields are used as a pump source in a short nonlinear crystal, the resulting second harmonic, as described by Eq. S2, can be expressed as

|  | $E_{2\omega}\left( \mathbf{r} \right)=E_{2\omega}^{\mathrm{co}}\left( \mathbf{r} \right)\exp\left[ -i\Phi_{2\omega}\left( \mathbf{r} \right) \right]$ $=\frac{i\kappa L}{2k_{2}}\left[ E_{\omega}^{\mathrm{co}}\left( \mathbf{r} \right) \right]^{2}\exp\left[ -i2\Phi_{\omega}\left( \mathbf{r} \right) \right].$ | (S4) |
| --- | --- | --- |

where $E_{\omega}^{\mathrm{co}}\left( \mathbf{r} \right)$ and $E_{2\omega}^{\mathrm{co}}\left( \mathbf{r} \right)$ are the fully coherent counterparts at the fundamental frequency and second harmonic in the complex field envelope form, respectively; $\Phi_{\omega}\left( \mathbf{r} \right)$ and $\Phi_{2\omega}\left( \mathbf{r} \right)$ are the random phase that defines the spatial correlation of the beams. For a Gaussian random phase $\Phi_{2\omega}\left( \mathbf{r} \right)$ with the zero mean in the synthesized second harmonic, the corresponding second-order correlation function is given by

|  | $\left\langle\Phi_{2\omega}\left( \mathbf{r}_{\mathbf{1}} \right)\Phi_{2\omega}\left( \mathbf{r}_{\mathbf{2}} \right) \right\rangle=exp\left[ -\frac{\left( \mathbf{r}_{\mathbf{1}}\mathbf{-}\mathbf{r}_{\mathbf{2}} \right)^{2}}{2\sigma_{\mu,2\omega}^{2}} \right],$ | (S5) |
| --- | --- | --- |

where $\sigma_{\mu,2\omega}$ is the coherence width at the second harmonic. The contribution from such random phase to the coherence function of the structured light beams can be derived by,

|  | $\Gamma_{2\omega}^{r}\left( \mathbf{r}_{\boldsymbol{1}}\boldsymbol{,}\mathbf{r}_{\boldsymbol{2}} \right)=\left\langle\exp\left[ i\left( \Phi_{2\omega}\left( \mathbf{r}_{\mathbf{1}} \right)-\Phi_{2\omega}\left( \mathbf{r}_{\mathbf{2}} \right) \right) \right] \right\rangle=exp\left[ -\frac{1}{2}D_{\Phi}\left( \mathbf{r}_{\mathbf{1}}\mathbf{,}\mathbf{r}_{\mathbf{2}} \right) \right]$ $=\exp\left[ -\frac{1}{2}\left\langle\left( \Phi_{2\omega}\left( \mathbf{r}_{\mathbf{1}} \right)-\Phi_{2\omega}\left( \mathbf{r}_{\mathbf{2}} \right) \right)^{2} \right\rangle\right]=exp\left[ \left\langle\Phi_{2\omega}\left( \mathbf{r}_{\mathbf{1}} \right)\Phi_{2\omega}\left( \mathbf{r}_{\mathbf{2}} \right) \right\rangle\right]$ $=\exp\left[ \exp\left[ -\frac{\left( \mathbf{r}_{\mathbf{1}}\mathbf{-}\mathbf{r}_{\mathbf{2}} \right)^{2}}{2\sigma_{\mu,2\omega}^{2}} \right] \right]\approx exp\left[ 1-\frac{\left( \mathbf{r}_{\mathbf{1}}\mathbf{-}\mathbf{r}_{\mathbf{2}} \right)^{2}}{2\sigma_{\mu,2\omega}^{2}} \right].$ | (S6) |
| --- | --- | --- |

It is worth mentioning here that the derivation of the first row relies on the relationship between the statistical average of the phase difference and the structure function of the random process [2], while the derivation of the last row utilizes the power series expansion of the exponential function [3,4]. When analyzing the coherence associated with the random phase of the fundamental frequency beams, it becomes evident that, since the values of two phase, $\Phi_{\omega}\left( \mathbf{r} \right)$ and $\Phi_{2\omega}\left( \mathbf{r} \right)$, differ only by a factor of 2, appropriate substitution readily yields also the Gaussian correlation with $\sigma_{\mu,\omega}=2\sigma_{\mu,2\omega}$.

Supplementary Note 3: Nonlinear filtering with incoherent light sources


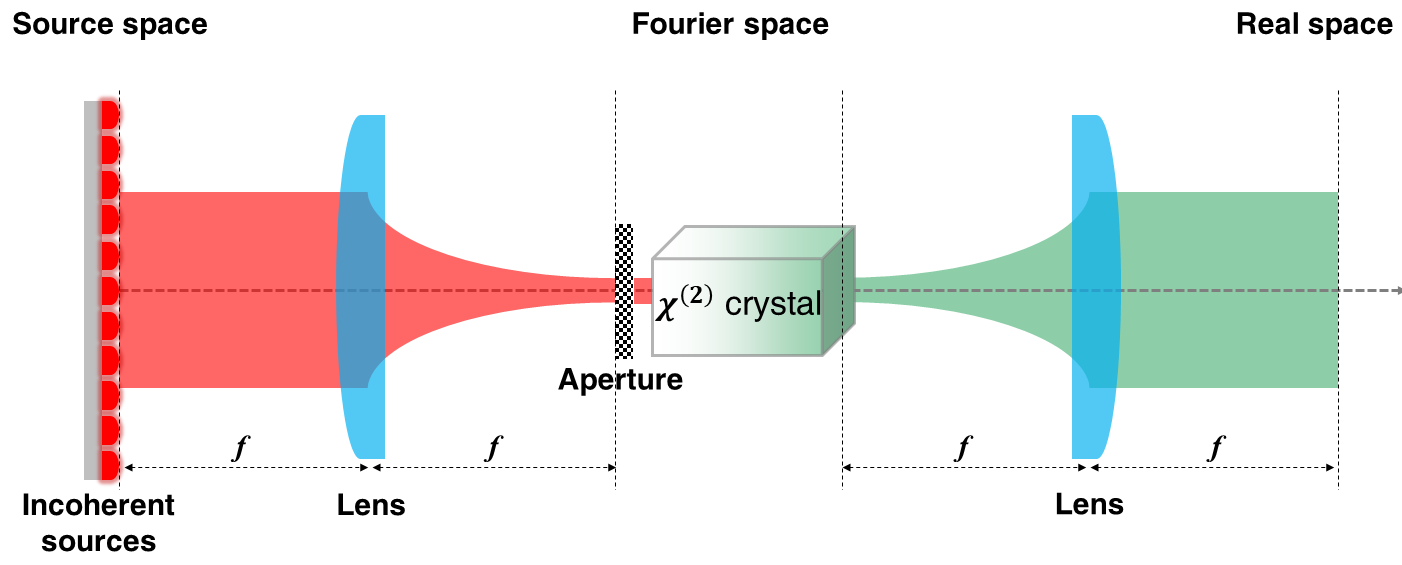


Fig. S1: **Schematic of nonlinear filtering**. A fully incoherent light source is propagated to its Fourier plane, where it undergoes filtering through an aperture before entering a nonlinear crystal. The filtered beams act as a pump to trigger the nonlinear interactions with the quadratic nonlinear photonic crystal. The resulting second harmonic emission is then analyzed in the crystal's far field.

In this section, we will formulate a theoretical model for nonlinear filtering with incoherent light sources, which will enhance our understanding of the nonlinear generation of incoherent structured light. As depicted in Fig. S1, the model comprises a 4*f* optical system, including a fully incoherent light source with intensity $I(\mathbf{r})$ positioned at the light source plane of the 4*f* system, a quadratic nonlinear crystal, and an aperture with a transmission function $T(\mathbf{r})$ located near the crystal’s incident plane at the Fourier plane of the 4*f* system.

This model can be interpreted as an incoherent light source that, upon passing through the first Fourier lens, generates a light field with specified coherence due to the van Cittert–Zernike theorem [4]. This light field then acts as a source to modulate the structured light field defined by the aperture's transmission function. The modulated light field is subsequently used as a pump source to generate the corresponding second harmonic under the weak interaction approximation, which is finally analyzed in the crystal's far field. Analogous to the derivation of linear filtering [5], obtaining the coherence function of the second harmonic beams in the real-space plane is straightforward as,

|  | $\Gamma_{2\omega}\left( \mathbf{r}_{\mathbf{1}}\mathbf{,}\mathbf{r}_{\mathbf{2}} \right)=I_{0}\int\int\int\int_{-\infty}^{\infty} I\left( \boldsymbol{\xi} \right)I\left( \boldsymbol{\eta} \right)\tilde{O}^{*}\left( \frac{\mathbf{r}_{\mathbf{1}}}{\lambda_{2}f}+\frac{\boldsymbol{\xi+\eta}}{\lambda_{1}f} \right)\tilde{O}\left( \frac{\mathbf{r}_{\mathbf{1}}}{\lambda_{2}f}+\frac{\boldsymbol{\xi+\eta}}{\lambda_{1}f} \right)d^{2}\boldsymbol{\xi}d^{2}\boldsymbol{\eta,}$ | (S7) |
| --- | --- | --- |

where $I_{0}$ is the normalized constant; $\tilde{O}\left( \mathbf{r} \right)$ is the Fourier transform of$\left[ T(\boldsymbol{\rho}) \right]^{2}$; $\lambda_{1}$ and $\lambda_{2}$ are the wavelength of the first and second harmonics; $f$ is the focal length of the lenses.

Eq. S7 can be physically interpreted as the coherence of the second harmonic arising from a linear superposition of multiple independent coherent modes, each displaced transversely by $\delta=\left( \boldsymbol{\xi+\eta} \right)/{\lambda_{1}f}$. The contribution of each displaced mode is weighted by $\alpha\left( \delta\right)=I\left( \boldsymbol{\xi} \right)I\left( \boldsymbol{\eta} \right)$. Thus, in this model, a Gaussian Schell-model source subjected to cubic phase modulation can be equivalently represented by a Gaussian intensity distribution for the source and a cubic phase transmission function for the aperture. Here, the function $\tilde{O}\left( \mathbf{r} \right)$ corresponds to each independent Airy mode, while the weight $\alpha\left( \delta\right)$ is characterized by a Gaussian distribution.

References

1. Boyd, R. W. *Nonlinear Optics*. (Academic Press, 2008).
2. Goodman, J. W. *Statistical Optics*. (Wiley, 2015).
3. Shirai, T. & Wolf, E. Coherence and polarization of electromagnetic beams modulated by random phase screens and their changes on propagation in free space. *J. Opt. Soc. Am. A* **21**, 1907–1907 (2004).
4. Zernike, F. The concept of degree of coherence and its application to optical problems. *Physica* **5**, 785 (1938).
5. Saleh, A., Abouraddy, A. F., Sergienko, A. V. & Teich, M. C. Duality between partial coherence and partial entanglement. *Phys. Rev. A* **62**, 043816, (2000).

Video 1.

**Nonlinear synthesis of object-induced coherence**. **a**, The patterns of the SLM. **b**, Each frame of the second harmonic speckle. **c**, The imaging induced by the synthesized coherence.

Video 2.

**Nonlinear generation of incoherent vortex beams from** $\mathcal{l}_{\boldsymbol{\omega}}\boldsymbol{\hbar}\boldsymbol{=1\hbar}$ **to** $\mathcal{l}_{\boldsymbol{2\omega}}\boldsymbol{\hbar}\mathbf{=2}\boldsymbol{\hbar}$. **a**, Statistical averages of the measured interferogram for the incoherent vortex beams at the fundamental frequency by the wavefront folding interferometer. **b**, The Fourier transform of the interferogram, showcasing the superposition of the -1^st^, 0, and 1^st^ diffraction orders due to the tilting between the two fields arising from each arm of the interferometer. **c**, Statistical averages of the far-field correlation function at the fundamental frequency upon an inverse Fourier transform for the -1^st^ diffraction order. **d-f**, The same measurements for the synthesized second harmonic incoherent vortex beams.

Video 3.

**Nonlinear generation of incoherent vortex beams from** $\mathcal{l}_{\boldsymbol{\omega}}\boldsymbol{\hbar}\boldsymbol{=2\hbar}$ **to** $\mathcal{l}_{\boldsymbol{2\omega}}\boldsymbol{\hbar}\boldsymbol{=4\hbar}$. **a**, Statistical averages of the measured interferogram for the incoherent vortex beams at the fundamental frequency by the wavefront folding interferometer. **b**, The Fourier transform of the interferogram, showcasing the superposition of the -1^st^, 0, and 1^st^ diffraction orders due to the tilting between the two fields arising from each arm of the interferometer. **c**, Statistical averages of the far-field correlation function at the fundamental frequency upon an inverse Fourier transform for the -1^st^ diffraction order. **d-f**, The same measurements for the synthesized second harmonic incoherent vortex beams.

Video 4.

**Nonlinear generation of incoherent Airy beams. a-c**, The patterns of the SLM corresponding to the measurements for d-f respectively. **d-f**, Statistical averages of the measured two-dimensional second harmonic incoherent Airy beams for various coherence width $\sigma_{\mu}=0.5w_{0}, w_{0}, 5w_{0}$
